# Regulation of age-dependent expression patterns of five transcription factors in *Larix kaempferi*

**DOI:** 10.48130/FR-2023-0018

**Published:** 2023-07-31

**Authors:** Xiangyi Li, Dongxia Cheng, Liwang Qi, Jinwei Zhan, Wanfeng Li

**Affiliations:** 1 State Key Laboratory of Tree Genetics and Breeding, Key Laboratory of Tree Breeding and Cultivation, National Forestry and Grassland Administration, Research Institute of Forestry, Chinese Academy of Forestry, Beijing 100091, People’s Republic of China; 2 State-owned Dagujia Forestry Farm in Qingyuan Man Autonomous County, Liaoning 113305, People’s Republic of China

**Keywords:** APETALA 2, Gene expression, Larch, MADS-box, Tree, Post-transcriptional regulation

## Abstract

To reveal the regulatory mechanisms underlying age-dependent expression patterns, we characterized seven age-related genes, *LaDAL1*, *LaAGL2-2*, *LaAGL2-3*, *LaAGL11*, *LaSOC1-1*, *LaAP2-1*, and *LaAP2-2* in terms of transcription and intron splicing in *Larix kaempferi*. Based on the exon–intron structures, we quantified the pre-mRNA levels and mature mRNA levels of these seven genes using quantitative reverse transcription polymerase chain reaction experiments. We found that the pre-mRNA levels manifested age-related patterns, indicating that their transcription was primarily regulated by age. By comparing the increasing or decreasing rates of the pre-mRNA and spliced mRNA levels, we found that their splicing efficiencies also changed with age. These results clearly show that both pre-mRNA transcription and splicing of five age-related genes are regulated by age, indicating that age-dependent expression patterns are controlled at both transcriptional and post-transcriptional levels, and unveiling the underlying regulatory molecular mechanisms should focus on the transcription factors, epigenetic regulation, and RNA splicing. These data provide new insights into the age-mediated regulation of gene expression in woody perennials in terms of longevity.

## Introduction

Age-related gene expression is essential for the growth and development of woody perennials, including the control of the growth rate^[1]^, rooting ability^[2]^, body architecture formation^[3]^, wood formation^[4−11]^, the juvenile period^[12,13]^, and reproductive phase changes^[12,13]^. As trees age, many genes change their expression^[14−19]^, indicating their functions and roles vary with development. Conifers are long-lived woody perennials that require a long time to bloom^[20,21]^. The conifer *Larix kaempferi* is characterized by rapid growth and is appreciated for its economic value. The juvenile period of *L. kaempferi* is about 10 years, which limits genetic improvement by sexual hybridization and production of improved varieties. In addition, to maintain the excellent traits of *L. kaempferi* mother trees, vegetative reproduction is commonly used. However, it often takes a long time to identify the excellent traits, and when these traits are identified, the rooting ability of cuttings decreases greatly. Thus, investigating the mechanisms underlying the effects of aging on *L. kaempferi* development is of theoretical and practical importance.

In our previous study, a comparative transcriptomic analysis of the uppermost main stems of 1-, 2-, 5-, 10-, 25-, and 50-year-old active *L. kaempferi* trees was performed to reveal the molecular aspects of aging on wood formation^[5]^ and phase change^[16]^, and it was found that many genes alter their expression with age^[16]^. Furthermore, seven transcription factors have age-related expression patterns^[15]^. *L*. *kaempferi DEFICIENS-AGAMOUS-LIKE 1* (*LaDAL1*/*LaAGL2-1*), *L. kaempferi* *AGAMOUS-Like 2-2* (*LaAGL2-2*), *LaAGL2-3*, and *L. kaempferi SUPPRESSOR OF OVEREXPRESSION OF CONSTANS 1-1* (*LaSOC1-1*) show increased transcription at 5 years, *LaAGL11* shows increased transcription at 3 years, and *L. kaempferi* *APETALA 2-2* (*LaAP2-2*) shows decreased transcription at 1 year^[15]^. Further analysis reveals that both *LaDAL1* and *LaAP2-1* have age-dependent expression patterns in both active and dormant stages^[14]^. Similarly, age-dependent patterns have also been revealed for homologs of *LaDAL1* in *Picea abies*^[22]^, *Pinus koraiensis*^[18]^, and *Pinus tabuliformis*^[17]^, indicating that these *DAL1* homologs have conservative roles and can be used to explore the age-mediated control of tree growth and development by studying their regulatory mechanisms. However, these studies have only detected the age-dependent expression patterns, and further studies are needed to understand how age regulates their expression *via* the underlying mechanisms.

**Figure 1:**
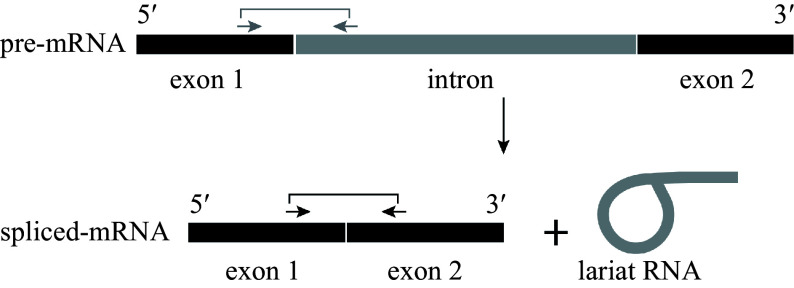
Process of mRNA splicing. RNA splicing involves cutting an intron (gray) from pre-mRNA and joining together the two neighboring exons (black). The spliced exons form the functional RNA, and the intron is usually degraded. Arrows indicate the positions of the primers used to detect the pre-mRNA (gray) and spliced mRNA (black).

Transcription and precursor messenger RNA (pre-mRNA) splicing are critical in the control of gene expression. In eukaryotes, pre-mRNA is transcribed by RNA polymerase II in a complicated process^[23]^. Genes in higher eukaryotes usually contain several introns. These introns are often of considerable length, sometimes extending to many thousands of bases. As such, they can account for 90% of the length of a typical pre-mRNA^[24]^. The vast majority of eukaryotic introns are not self-splicing, and the formidable task of identifying and splicing together exons among all the intronic RNA is performed by the spliceosome, a large ribonucleoprotein machine^[25,26]^. After splicing, the two concomitant exons are joined, and the intron is released as a lariat RNA^[25,27]^ (Fig. 1). In previous studies, various age-dependent expression patterns have been revealed at the mature mRNA level^[15]^. However, it remains unclear which step from pre-mRNA transcription to splicing is regulated by age.

Here, we hypothesized that the expression of age-related genes may be regulated at the levels of transcription and mRNA processing. To gain further insights into the age-mediated regulation of gene expression in *L. kaempferi*, we studied pre-mRNA transcription and splicing efficiency of seven age-related genes during tree aging. Specific primers were designed to detect pre-mRNA and spliced mRNA changes in trees of different ages. Comparisons of pre-mRNA and spliced mRNA change fold can be instrumental in the analysis of splicing efficiency. Our results provide novel insights into the age-mediated regulation of gene expression in tree growth and development.

## Materials and methods

### DNA sequence analysis and primer design

The DNA sequences of age-related transcription factors were obtained from the *L. kaempferi* genome^[28]^. The gene structure was analyzed using Gene Structure Display Server 2.0^[29]^. Quantitative reverse transcription polymerase chain reaction (qRT-PCR) was used to examine the expression patterns of pre-mRNA and spliced mRNA. Specific primers were designed to detect pre-mRNA and spliced mRNA (www.ncbi.nlm.nih.gov/tools/primer-blast/, accessed on 20 March 2022) (Table 1 & Supplemental Table S1). For each gene, three pairs of primers were designed against the 5′ end, middle, and 3′ end.

**Table 1:** Polymerase chain reaction primers for the amplification of age-related transcription factors.

Gene (accession: mRNA/DNA)	Primer	Sequence (5'-3')	Position	Size (bp)
*LaDAL1* (MN790744/WOXR02001943.1)	I	Forward-CGATGCAGAAGTGGCGCTAA	first exon	94
		Reverse-GTCAACAGCGCAAAGAAAGGA	first intron	
	II	Forward-GCTCTCAGTGCTGTGCGAT	first exon	249
		Reverse-CCGAGATCTTCCCCCAACAAA	fourth exon	
	III	Forward-AGACCAGATTGAGGAGCTTCG	fifth exon	90
		Reverse-GGGACGGAATAGCGTGCATTA	fifth intron	
	IV	Forward-GGTTGAGCTCCTTCAGCGAT	third exon	163
		Reverse-GCGAAGCTCCTCAATCTGGT	fifth exon	
	V	Forward-GTACTAACGGGCCTTGGGAT	seventh exon	172
		Reverse-TTCCAGCTTCAAAAGTGCCAAT	seventh intron	
	VI	Forward-ACGCAGGTGATGCTAGACCA	fifth exon	165
		Reverse-CCAAGGCCCGTTAGTACCAG	seventh exon	
*LaAGL2-2* (MN790745/WOXR02003181.1)	I	Forward-GAGTTTGCTAGTGCCGGGTA	first exon and first intron	207
		Reverse-GTTGGGGGAAGATCTGGGTC	first intron	
	II	Forward-GGGCTGCTGAAGAAAGCCTA	first exon	110
		Reverse-TTCATGCCGGCACTAGCAAA	second exon	
	III	Forward-GTTGGGGGAAGATCTGGGTC	fourth exon	242
		Reverse-TGCGTTGTGTCGTATTTAGGTC	fourth intron	
	IV	Forward-TGCAGCAACTCGAACATCAAC	fourth exon	76
		Reverse-TGGCCTAGCATAACCTGCG	fifth exon	
	V	Forward-AATTCAAGCCTCCCGACTGT	sixth exon	207
		Reverse-TCCGGGGACTACATATTGGC	sixth intron	
	VI	Forward-TGCTCTCTTACACCCGCAAC	sixth exon	141
		Reverse-CCACCACCCTTGCACGTAT	seventh exon	
*LaAP2-1* (MN790757/WOXR02007023.1)	I	Forward-CCCCGGAGTTCTGAGGAAAC	first exon	197
		Reverse-TTGCTAGAGGCCTCGTGTTC	first intron	
	II	Forward-GCTCGCAATATCGTGGAGTG	first exon	117
		Reverse-TAGCAGCGGCATGAGCAGTA	third exon	
	III	Forward-CAGCTATCAAGTTTCGAGGCG	fourth exon	200
		Reverse-ACTGCCATCCAAATGACTACC	fourth intron	
	IV	Forward-TTCGAGGCGTTGAAGCTGAT	fourth exon	131
		Reverse-TCCACGAGAGAAACCAGTGC	fifth exon	
	V	Forward-CTGAAGCTCACATGAGGGAGG	ninth exon	158
		Reverse-TCCGCTCAGTCCATCTTTATGC	ninth intron	
	VI	Forward-CCTGACCATCTGGGTAACTGT	ninth exon	159
		Reverse-TACTGGAGTTGTTGGTCCGC	tenth exon	

### Plant materials

In our previous work, we found that the mature mRNA levels of *LaDAL1*, *LaAGL2-2*, *LaAGL2-3*, *LaAGL11*, and *LaSOC1-1* increased from 1 to 7 years, whereas the mature mRNA levels of *LaAP2-1* and *LaAP2-2* decreased from 1 to 5 years^[15]^. Based on these results, in this work, we used 1-, 3-, 5- and 13-year-old active *L. kaempferi* trees that were collected on 4 July 2019^[15]^. These trees were grown from seeds and included the vegetative and reproductive phases of *L. kaempferi*. They were collected from a seed orchard in Dagujia (42°22′ N, 124°51′ E), Liaoning Province, in Northeast China. During sampling, main stems or lateral branches from the upper crowns were collected. After removal of buds or needles, the left stems from at least six trees from each age category were pooled, frozen in liquid nitrogen, and stored at −80 °C until RNA extraction.

### RNA extraction, cDNA synthesis, and qRT-PCR

These experiments were carried out as previously described^[15]^. *L. kaempferi* *translation elongation factor-1 alpha 1* (*LaEF1A1*) (GenBank accession no. JX157845), which was used as the internal control, is expressed stably during tree aging^[5]^, and the primers 5′-GACTGTACCTGTTGGTCGTG-3′ and 5′-CCTCCAGCAGAGCTTCAT-3′ were used^[30]^. The relative expression ratio was expressed using the 2^−ΔΔCt^ method^[31]^. The sample with the minimum or maximum age was used for normalization and was set to a value of 1. The qRT-PCR was performed with four technical replicates, and the data are shown as the mean ± standard deviation.

## Results and discussion

### Super-long introns and CpG islands exist in some age-related genes

**Figure 2:**
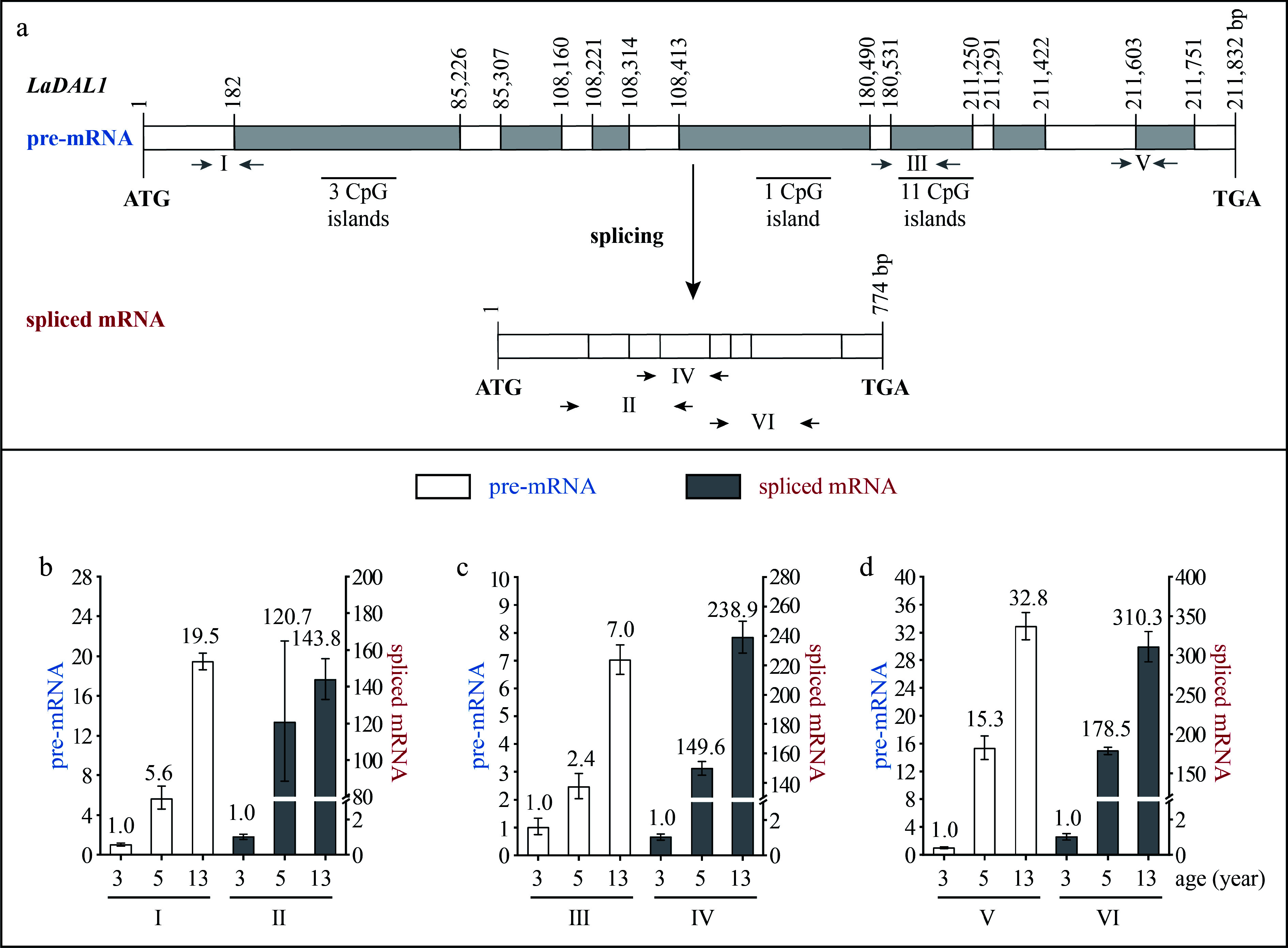
Structure and expression pattern of *LaDAL1*. (a) Schematic representation of the gene structure of *LaDAL1*. White indicates exon; gray indicates intron; gray arrows indicate the positions of the primers used to measure the pre-mRNA; and black and horizontal arrows indicate the positions of the primers used to measure the spliced mRNA. After the prediction of DNA methylation, the CpG islands were revealed. (b)–(d) Expression patterns of *LaDAL1* pre-mRNA and spliced mRNA during tree aging detected by three different primer pairs. The lateral branches of 3-, 5-, and 13-year-old active *Larix kaempferi* trees (n ≥ 6, sampled on 4 July 2019) were used to examine the expression patterns, which were assayed by qRT-PCR with *LaEF1A1* as the internal control. The capitalized Roman numerals (I–VI) in panels (a)–(d) represent the different primers. The *p*-values of the differences between 5- and 3-year-old trees were calculated. One-way ANOVA Duncan’s test was used for statistical analysis.

The *LaDAL1* DNA sequence was 211,832 bp in length and contained eight exons and seven introns, with an open reading frame (ORF) of 774 bp, which encoded 257 amino acids (aa) (Fig. 2a). The exon length ranged from 42 to 182 bp, and the intron length ranged from 92 to 85,043 bp. The first, second, fourth, and fifth introns were more than 10 kb in length and were super-long introns (Fig. 2a). Fifteen CpG islands were predicted in the super-long introns (Fig. 2a).

**Figure 3:**
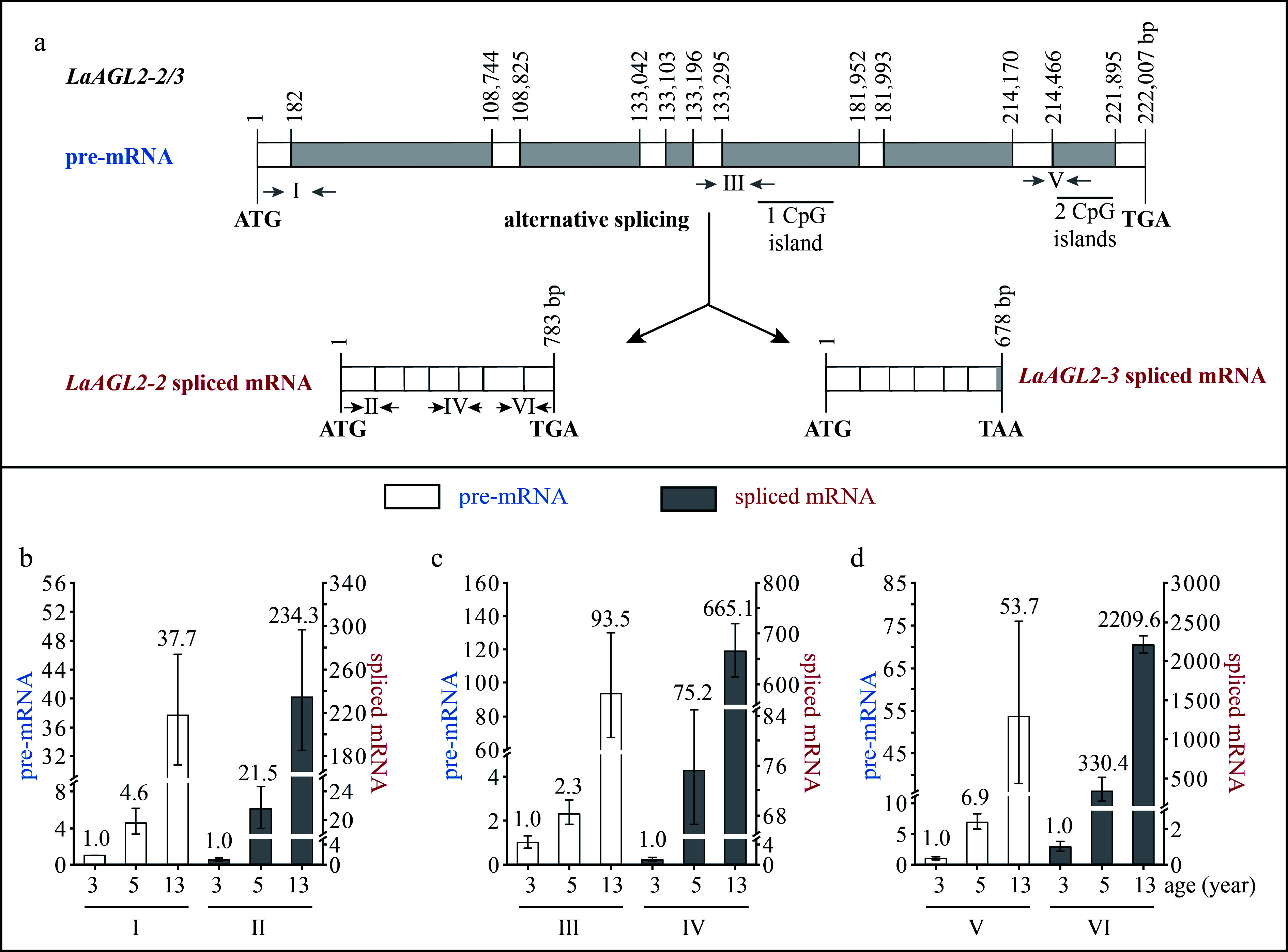
Structure and expression pattern of *LaAGL2-2/3*. (a) Schematic representation of the gene structure of *LaAGL2-2/3*. White indicates exon; gray indicates intron; gray arrows indicate the positions of the primers used to measure the pre-mRNA; and black and horizontal arrows indicate the positions of the primers used to measure the spliced mRNA. After the prediction of DNA methylation, the CpG islands were revealed. (b)–(d) Expression patterns of *LaAGL2-2/3* (b, c) and *LaAGL2-2* (d) pre-mRNA and spliced mRNA during tree aging detected by three different primer pairs. The lateral branches of 3-, 5-, and 13-year-old active *Larix kaempferi* trees (n ≥ 6, sampled on 4 July 2019) were used to detect the expression patterns, which were assayed by qRT-PCR with *LaEF1A1* as the internal control. The capitalized Roman numerals (I–VI) in panels (a)–(d) represent the different primers. The *p*-values of the differences between 5- and 3-year-old trees were calculated. One-way ANOVA Duncan’s test was used for statistical analysis.

*LaAGL2-2* and *LaAGL2-3* were two splice variants, because their transcript sequences were mapped to the same DNA sequence. This DNA sequence was 222,007 bp in length and contained seven exons and six introns (Fig. 3a). The transcript sequence of *LaAGL2-2* was 783 bp in length, encoding a polypeptide of 260 aa, and that of *LaAGL2-3* was 678 bp in length, encoding a polypeptide of 225 aa (Fig. 3a). The *LaAGL2-3* spliced mRNA retained part of the sequence of its sixth intron in which there was a termination codon. The first, second, fourth, and fifth introns were super-long introns, and three CpG islands were predicted in the introns (Fig. 3a).

**Figure 4:**
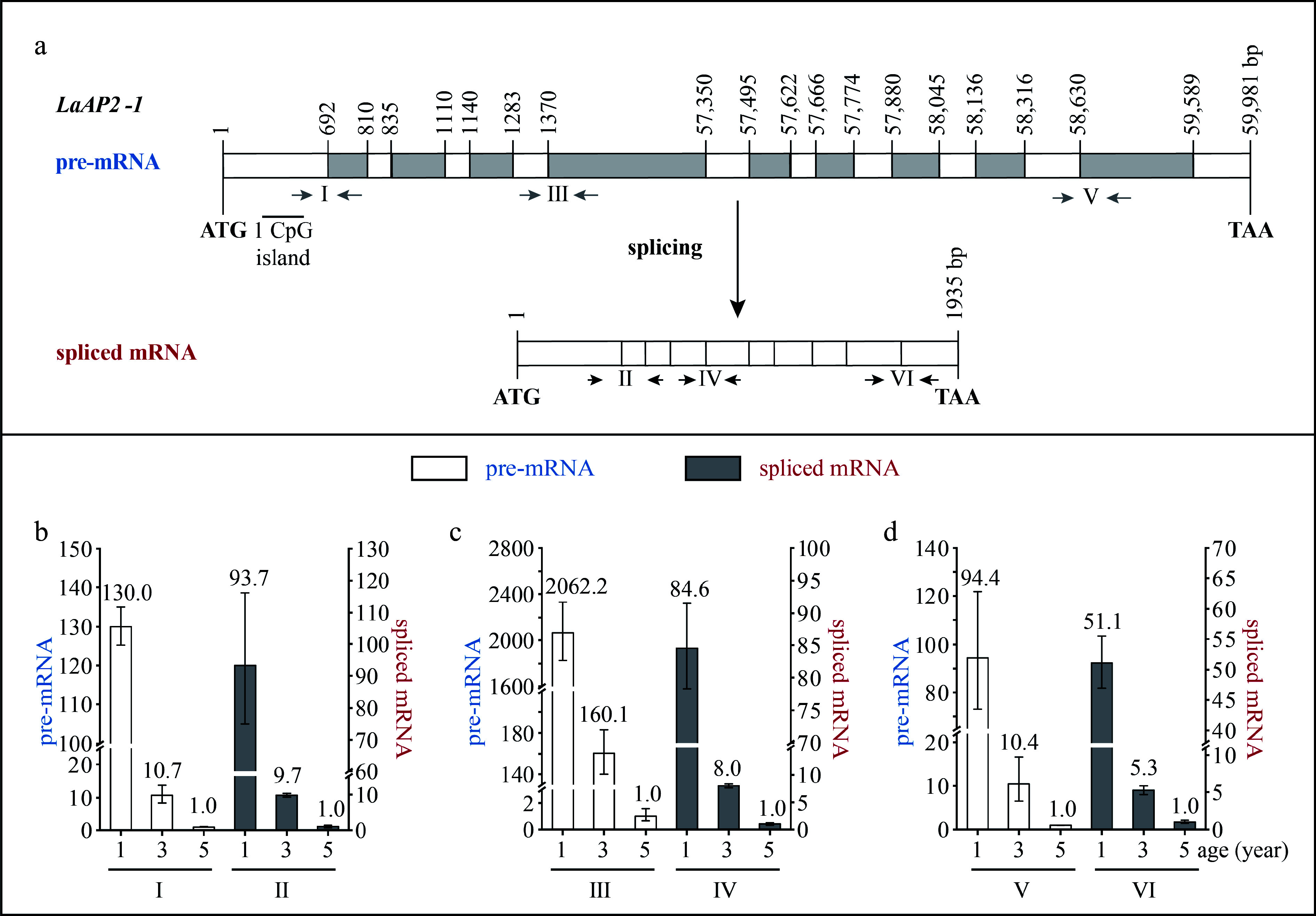
Structure and expression pattern of *LaAP2-1*. (a) Schematic representation of the gene structure of *LaAP2-1*. White indicates exon; gray indicates intron; gray arrows indicate the positions of the primers used to measure the pre-mRNA; and black and horizontal arrows indicate the positions of the primers used to measure the spliced mRNA. After the prediction of DNA methylation, the CpG islands were revealed. (b)–(d) Expression patterns of *LaAP2-1* pre-mRNA and spliced mRNA during tree aging detected by three different primer pairs. The lateral branches of 1-, 3-, and 5-year-old active *Larix kaempferi* trees (n ≥ 6, sampled on 4 July 2019) were used to detect the expression patterns, which were assayed by qRT-PCR with *LaEF1A1* as the internal control. The capitalized Roman numerals (I–VI) in panels (a)–(d) represent the different primers.

The *LaAP2-1* DNA sequence was 59,981 bp in length and contained ten exons and nine introns, with an ORF of 1,935 bp, which encoded 644 aa (Fig. 4a). The exon length ranged from 26 to 692 bp, and the intron length ranged from 107 to 55,979 bp. The fourth intron was a super-long intron, and one CpG island was predicted in the exon (Fig. 4a).

Based on these data, we speculated that the existence of the super-long introns and CpG islands in these age-related genes may control their transcription by affecting site recognition and splicing by the spliceosome. Notably, in the *P. tabuliformis DAL1* (*PtDAL1*) DNA sequence, super-long introns and CpG islands also exist, and two segments at the 5′ end of the first ultra-long intron show a gradual decline of CHG methylation with increasing age, which is negatively correlated with its expression^[32]^, indicating that increased *PtDAL1* transcription during tree aging may be controlled by CHG methylation in this intron. These results not only support our speculation but also provide new insights on the mechanism underlying the formation of age-dependent expression patterns.

### Transcription of age-related genes is regulated by age

The *LaDAL1* pre-mRNA level in 5-year-old trees was about 5.6-times higher as detected by primer I (Fig. 2b), 2.4-times higher as detected by primer III (Fig. 2c, *p* < 0.05), and 15.3-times higher as detected by primer V (Fig. 2d, *p* < 0.05) than that in 3-year-old trees. Compared with 5-year-old trees, a higher level of *LaDAL1* pre-mRNA was detected in 13-year-old trees (Fig. 2b−d ). The *LaAGL2-2/3* pre-mRNA level in 5-year-old trees was about 4.6-times higher as detected by primer I (Fig. 3b, *p* < 0.05) and 2.3-times higher as detected by primer III (Fig. 3c, *p* < 0.05) than that in 3-year-old trees. Compared with 5-year-old trees, a higher level of *LaAGL2-2/3* pre-mRNA was also detected in 13-year-old trees. The *LaAP2-1* pre-mRNA level in 1-year-old trees was about 103.0-times higher as detected by primer I (Fig. 4b, *p* < 0.05), 2,062.2-times higher as detected by primer III (Fig. 4c, *p* < 0.05), and 94.4-times higher as detected by primer V (Fig. 4d, *p* < 0.05) than that in 5-year-old trees.

Increased *LaAGL11* and *LaSOC1-1* pre-mRNA levels (Supplemental Figs S1 & S2), as well as a decreased *LaAP2-2* pre-mRNA level, were also detected (Supplemental Fig. S3). These results showed that the pre-mRNAs of these seven age-related genes also have age-dependent expression patterns and that the age regulation of their transcription does occur. Notably, many CpG islands were predicted in the introns of five genes (*LaDAL1*, *LaAGL11*, *LaAGL2-2/3*, and *LaSOC1*) (Figs 2a & 3a, Supplemental Figs S1a & S2a), which are highly expressed in the reproductive stage. However, no CpG islands were predicted in the introns of two genes (*LaAP2-1* and *LaAP2-2*) (Fig. 4a & Supplemental Fig. S3a), whose transcript levels decreased with tree aging, indicating that other regulatory mechanisms of the transcription of these age-related genes exist in addition to DNA methylation^[32]^ and that age may control the expression of these up- and down-regulated genes with different mechanisms.

### Pre-mRNA splicing may contribute to the formation of age-dependent expression patterns

Pre-mRNA is the initial product of transcription, and its level can be detected when the primer used for qRT-PCR is designed within the intron, which is removed when pre-mRNA is extensively edited by splicing. Splicing is a process in which pre-mRNA is used as a substrate to produce spliced mRNA under the action of the spliceosome^[33]^. In this process, the splicing efficiency determines the production rate of spliced mRNA. Based on this, we measured the pre-mRNA levels of seven age-related genes and their splicing efficiencies.

The levels of *LaDAL1* pre-mRNA and spliced mRNA increased with age at different rates (Fig. 2b−d). For example, the *LaDAL1* pre-mRNA level in 5-year-old trees was about 5.6-times higher than that in 3-year-old trees (Fig. 2b, *p* < 0.05), whereas the spliced mRNA level in 5-year-old trees was about 120.7-times higher than that in 3-year-old trees (Fig. 2b, *p* < 0.05). Differences in the increasing rates in *LaDAL1* pre-mRNA and spliced mRNA levels also existed in 13- and 3-year-old trees. Compared with pre-mRNA, spliced mRNA showed a higher increase in its level in 5- and 13-year-old trees (Fig. 2b−d). Differences in the increasing rates in spliced mRNA levels were also detected in *LaAGL2-2*/*3* (Fig. 3b−d) and *LaAGL11* (Supplemental Fig. S1b−d). These results indicate that the production of spliced mRNA from the pre-mRNA may be enhanced with tree aging.

The levels of *LaAP2-1* pre-mRNA and spliced mRNA decreased with age at different rates (Fig. 4b−d). For example, the *LaAP2-1* pre-mRNA level in 1-year-old trees was about 2,062.2-times higher than that in 5-year-old trees (Fig. 4b, *p* < 0.05), whereas the spliced mRNA level in 1-year-old trees was about 84.6-times higher than that in 5-year-old trees (Fig. 4b, *p* < 0.05). Differences in the decreasing rate in *LaAP2-1* pre-mRNA and spliced mRNA levels were also detected in 3- and 5-year-old trees. Compared with spliced mRNA, pre-mRNA showed a smaller decrease in its level in 3- and 5-year-old trees (Fig. 4b−d), indicating that the production of *LaAP2-1* spliced mRNA from its pre-mRNA may be reduced in 3- and 5-year-old trees.

During tree aging, a greater increase in the *LaAGL2-2* transcript level was detected compared to that in the *LaAGL2-3* transcript level, because the *LaAGL2-2* and *LaAGL2-3* spliced mRNA levels in 13-year-old trees were about 234.3-times higher than those in 3-year-old trees as detected by primer II (Fig. 3b, *p* < 0.05), whereas the *LaAGL2-2* spliced mRNA level in 13-year-old trees was about 2209.6-times higher than that in 3-year-old trees as detected by primer VI (Fig. 3d, *p* < 0.05), which can only amplify *LaAGL2-2* (Fig. 3a). These data indicate that the alternative splicing of *LaAGL2-2/3* is controlled by age. It has been shown that light-mediated^[34]^ and temperature-mediated^[35]^ pre-mRNA alternative splicing in plants can regulate gene expression to promote morphogenesis and stress resistance. The role and significance of the alternative splicing of *LaAGL2-2/3* during aging require further study.

## Conclusions

Based on these data, we conclude that age affects the pre-mRNA transcription of seven age-related genes. Furthermore, our findings show that pre-mRNA splicing plays a role in controlling *LaDAL1*, *LaAGL11*, *LaAGL2-2/3*, and *LaAP2-1*, and their increased or decreased spliced mRNA levels during tree aging result from the increased or decreased transcription and the enhanced or reduced pre-mRNA splicing efficiency, respectively.

## SUPPLEMENTARY DATA

Supplementary data to this article can be found online.
