# Supplementary material for: Regulation of age-dependent expression patterns of five transcription factors in Larix kaempferi
Source: For Res (Fayettev). 2023 Jul 31;3:18. doi: 10.48130/FR-2023-0018 (PMC11524251; doi:10.48130/FR-2023-0018)
Supplement: Supplementary file 1 — Supplementary data to this article can be found online. [file FR-2023-0018-S1.zip › 10.48130_FR-2023-0018-Suppl-TableS1.docx]

**Table S1. Three age-related Larix kaempferi transcription factors and their qRT-PCR primers.**

| Gene (accession: mRNA/DNA) | Primer | Sequence (5'-3') | Position | Size (bp) |
| --- | --- | --- | --- | --- |
| *LaAGL11* (MN790752/WOXR02007254.1) | Ⅰ | Forward-AAGCTTACGAGCTCTCGGTG | first exon | 245 |
|  |  | Reverse-AAGTAACGGCCGGGTTATGG | first intron |  |
|  | Ⅱ | Forward-CCCGAGAGGGAAGCTGTATG | first exon | 101 |
|  |  | Reverse-CGGTTGCATTACTTCTGCCA | second exon |  |
|  | Ⅲ | Forward-TGAGTTGGAGAGCCAAGTTGA | fourth exon | 116 |
|  |  | Reverse-ACCTTTGAAAAGCACACATACACA | fourth intron |  |
|  | ⅠV | Forward-AGTTGGGGGAGGGTTTGGAAT | fourth exon | 96 |
|  |  | Reverse-TCTTCTGTGCTCGTATATTGCTCA | fifth exon |  |
|  | V | Forward-AAGCATTGATGGATCAAATAGAACT | fifth exon | 73 |
|  |  | Reverse-AGGTAAGGGAGGAATTGCAAAG | fifth intron |  |
|  | VⅠ | Forward-TGCAAGAGGTAGATGGTTGGG | fifth exon | 77 |
|  |  | Reverse-AGTCTCCACTTCATTGTGTTGC | sixth exon |  |
| *LaSOC1-1* (MN790747/WOXR02000999.1) | Ⅰ | Forward-AGGGTGTATTGCTTGGCCTT | 5' UTR first exon | 154 |
|  |  | Reverse-TCACAGGGTCTCAAGTCAGG | 5' UTR first intron |  |
|  | Ⅱ | Forward-TGGTCTATCTTTGGAAGGGAAGT | 5' UTR first exon | 159 |
|  |  | Reverse-GAGCACGGAGAGCTCGTAAG | second exon |  |
|  | Ⅲ | Forward-AAGCTTACGAGCTCTCCGTG | second exon and second intron | 102 |
|  |  | Reverse-ACCATACATACCAGGGACTGGA | second intron |  |
|  | ⅠV | Forward-CCCCCAGAGGGAAGCTCTAT | second exon | 118 |
|  |  | Reverse-TGAGTATCTTGCTCTTTGGTTGT | third exon |  |
|  | V | Forward-TGATGGATCAAATACAACAACTTGA | sixth exon | 216 |
|  |  | Reverse-TTCCAATTTCAGTCTTGAAACGAG | sixth intron |  |
|  | VⅠ | Forward-AGCAGCAAGATTGGGTTTGG | sixth exon | 74 |
|  |  | Reverse-GCTGAATCTCTATTCCACTGGT | seventh exon |  |
| *LaAP2-2* (MN790758/WOXR02002646.1) | Ⅰ | Forward-TCAGAAGGCAACGACGGAAA | first exon | 169 |
|  |  | Reverse-TGATGGGCATGGGATTAGGC | first intron |  |
|  | Ⅱ | Forward-GGAAAAACCCCCGCCTGATA | first exon | 225 |
|  |  | Reverse-ACCTGTTTCCCACAATCCCA | second exon |  |
|  | Ⅲ | Forward-GCAGTCACAAACTTCGAGCC | seventh exon | 132 |
|  |  | Reverse-TGAGTTTATCATGCGCTCTTCT | seventh intron |  |
|  | ⅠV | Forward-CAGTCACAAACTTCGAGCCCA | seventh exon | 108 |
|  |  | Reverse-TTGAAGCGGACGTGCCAAG | eighth exon |  |
|  | V | Forward-ACATTCTCGACCAAGCGAGG | ninth exon | 250 |
|  |  | Reverse-CTGAAAAGTGACCAGGCAAGT | ninth intron |  |
|  | VⅠ | Forward-ACATTCTCGACCAAGCGAGG | ninth exon | 201 |
|  |  | Reverse-CAAGTCCAGCCTGGTGTCTT | tenth exon |  |
