# Supplementary material for: Regulation of age-dependent expression patterns of five transcription factors in Larix kaempferi
Source: For Res (Fayettev). 2023 Jul 31;3:18. doi: 10.48130/FR-2023-0018 (PMC11524251; doi:10.48130/FR-2023-0018)
Supplement: Supplementary file 1 — Supplementary data to this article can be found online. [file FR-2023-0018-S1.zip › 10.48130_FR-2023-0018-Suppl-FigureS1.docx]

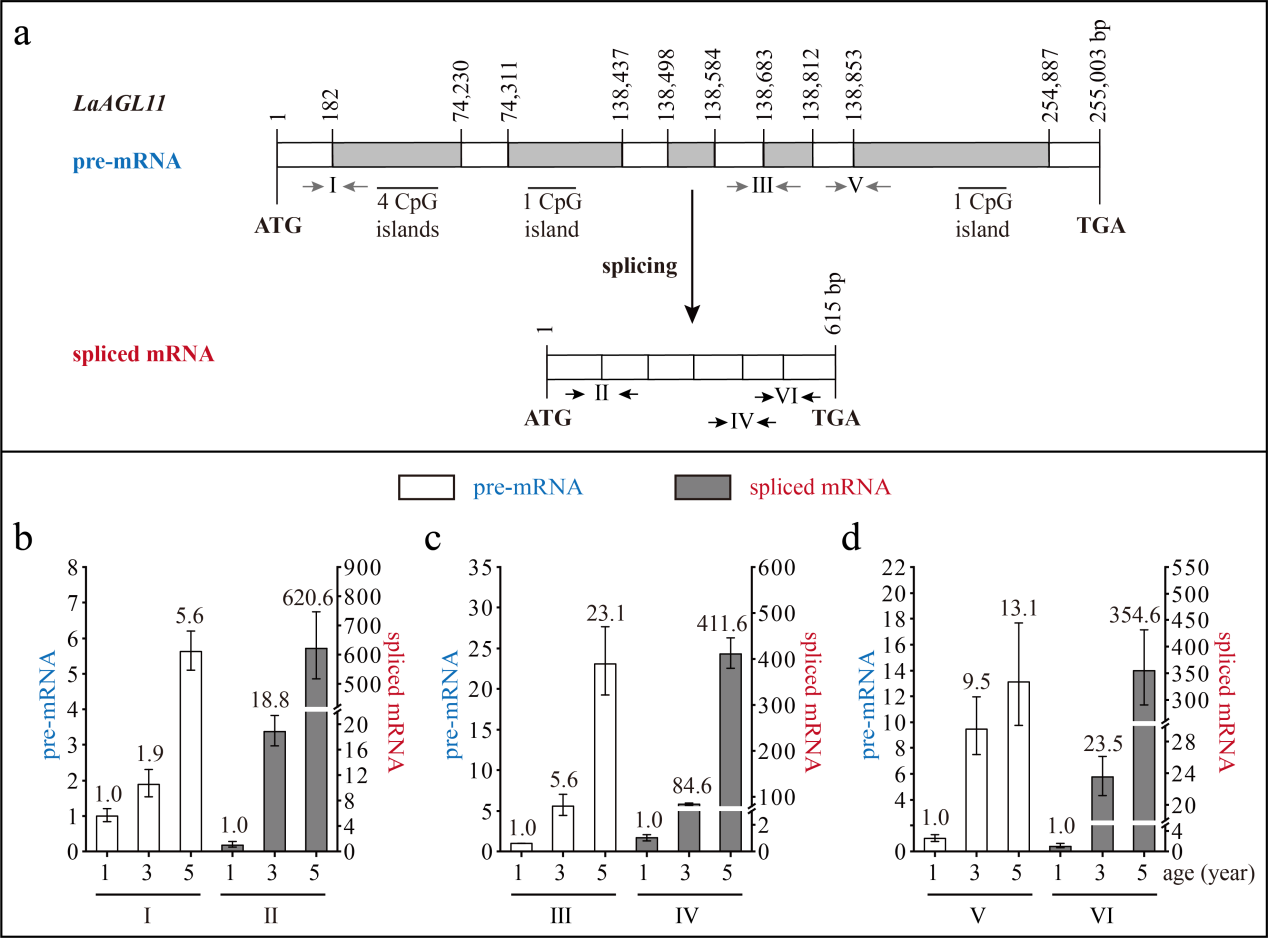


**Fig. S1** The structure and expression pattern of *LaAGL11*. (a) Schematic representation of the genomic structure of *LaAGL11*. White indicates exon; grey indicates intron; grey arrows indicate the positions of the primers used to measure the pre-mRNA; black arrows indicate the positions of the primers used to measure the spliced mRNA. (b-d) Expression patterns of *LaAGL11* pre-mRNA and spliced mRNA during tree aging detected by three different primer pairs. The lateral branches of 1-, 3-, and 5-year-old active *Larix kaempferi* trees (n ≥6, sampled on 4 July 2019) were used to detect the expression patterns, which were assayed by qRT-PCR with *LaEF1A1* as the internal control. The capitalized Roman numerals (Ⅰ-Ⅵ) in Fig. a-d represent the different primers. The *p*-value was calculated between 3- and 1-year-old trees. One-way ANOVA Duncan’s test was used for statistical analysis.
